# Sex-Dependent Expression of Caveolin 1 in Response to Sex Steroid Hormones Is Closely Associated with Development of Obesity in Rats

**DOI:** 10.1371/journal.pone.0090918

**Published:** 2014-03-07

**Authors:** Rajib Mukherjee, Sang Woo Kim, Myung Sook Choi, Jong Won Yun

**Affiliations:** 1 Department of Biotechnology, Daegu University, Kyungsan, Republic of Korea; 2 Center for Food and Nutritional Genomics Research & Department of Food Science and Nutrition, Kyungpook National University, Daegu, Republic of Korea; Hosptial Infantil Universitario Niño Jesús, CIBEROBN, Spain

## Abstract

Caveolin-1 (CAV1) is a conserved group of structural membrane proteins that form special cholesterol and sphingolipid-rich compartments, especially in adipocytes. Recently, it has been reported that CAV1 is an important target protein in sex hormone-dependent regulation of various metabolic pathways, particularly in cancer and diabetes. To clarify distinct roles of CAV1 in sex-dependent obesity development, we investigated the effects of high fat diet (HFD) and sex steroid hormones on CAV1 expression in adipose tissues of male and female rats. Results of animal experiments revealed that estrogen (17-β-estradiol, E2) and androgen (dihydrotestosterone, DHT) had opposite effects on body weight gain as well as on the regulation of CAV1, hormone sensitive lipase (HSL) and uncoupling protein 1 (UCP1) in adipose tissues. Furthermore, sex hormone receptors and aromatase were differentially expressed in a sex-dependent manner in response to E2 and DHT treatments. *In vivo* data were confirmed using 3T3-L1 and HIB1B cell lines, where *Cav1* knock down stimulated lipogenesis but suppressed sex hormone receptor signaling proteins. Most importantly, co-immunoprecipitation enabled the identification of previously unrecognized CAV1-interacting mitochondrial or lipid oxidative pathway proteins in adipose tissues. Taken together, current data showed that CAV1 may play important preventive role in the development of obesity, with more prominent effects in females, and proved to be an important target protein for the hormonal regulation of adipose tissue metabolism by manipulating sex hormone receptors and mitochondrial oxidative pathways. Therefore, we can report, for the first time, the molecular mechanism underlying the effects of sex steroid hormones in the sex-dimorphic regulation of CAV1.

## Introduction

Recently, interest in factors that regulate adipose tissue function has increased due to concerns over excess fat consumption in Western diets, which may mainly contribute to obesity and other metabolic diseases [1]. In adipose tissues, caveolin proteins are abundantly expressed and play roles in vesicular transport, cholesterol homeostasis, and signal transduction [2]. Several lines of evidence have indicated that caveolins may be implicated in the pathogenesis of various diseases, including cancer, diabetes, atherosclerosis, heart failure, muscular dystrophy, and Alzheimer’s disease [2]–[5]. Caveolin-1 (CAV1) is the most important member of the caveolin family, which is a conserved group of structural membrane proteins that form special cholesterol and sphingolipid-rich compartments, especially in adipocytes and endothelial cells in which caveolae constitutes 20–30% of the total plasma membrane [6]–[8].

Previous studies using *Cav1*-null mice have demonstrated reduced whole body fat mass and adiponectin levels, elevated triglycerides (TG) and free fatty acids (FFA), as well as blunted responses to β_3_-adrenergic receptor stimulation [2], [4]. Unfortunately, previous reports on the response of CAV1 expression to different physiological conditions are contradictory. For example, Razani and Lisanti [4] reported reduced lipid accumulation in *Cav1* knockout (KO) mice along with a lean phenotype. Cohen et al. [9] demonstrated reduced lipolytic capacity in *Cav1* KO mice, and Fernandez-Real et al. [10] showed attenuated *Cav1* expression in visceral and subcutaneous white adipose tissue (WAT) of obese patients. Moreover, a recent report demonstrated that loss of *Cav1* alters mitochondrial function in adipose tissues [7]. The reduction of adipose tissue in *Cav1*-null mice can be attributed to altered lipid deposition and improper storage of lipids outside of the adipose tissue [11]. The lean phenotype of *Cav1*-null mice is primarily related to the inability of adipose tissue to store lipids, which consequently remain in the circulation and cause massive elevation of free fatty acids and hypertriglycerides [11], [12]. Despite this recent progress, the precise roles of CAV1 remain unclear, especially in the development of obesity. Furthermore, it is not well established whether or not CAV1 exerts its effects in a sex-dependent manner as well as in response to sex steroid hormones.

In our previous studies, we found that male rats are more susceptible to obesity when fed a HFD, and many proteins play roles in obesity resistance in different metabolic tissues [13]–[16]. In conjunction with the present data, an earlier report demonstrated the anti-obesity effects of CAV1, as evidenced by reduced *Cav1* expression in visceral adipose tissue of obese patients [10]. Therefore, these data led us to hypothesize that CAV1 may play a protective role in diet-induced obesity, which strongly contradicts the lean phenotype observed in *Cav1*-deficient animal models reported by other investigators [7], [8], [17].

Sex steroid hormones and their receptors are well-known regulators of several aspects of metabolism, such as lipid and carbohydrate metabolism, and their impaired signaling is highly associated with the development of metabolic diseases [18]–[24]. In particular, estrogens has been shown to reduce the body weight of HFD-fed mice [25], reduce hepatic lipogenesis, improve insulin sensitivity [26], as well as promote the partitioning of free fatty acids toward oxidation [27].

In our previous studies, we found that numerous metabolic proteins in white adipose tissue (WAT), brown adipose tissue (BAT), and other metabolic tissues are regulated in a sex-dependent manner [28]–[32]. We believe that the different expression patterns of those proteins are the result of differential hormonal regulation, specifically estrogens and testosterone.

Previous evidence has demonstrated that CAV1 regulates sex hormone signaling by directly interacting with hormone receptors in the membrane, and hormones have been shown to modulate CAV1 expression in several types of cells [12], [33]–[35]. A variety of studies have raised the possibility that estrogens have more prominent effects than testosterone in diabetes and cancer [35]. Moreover, conflicting results have been obtained regarding the role of testosterone in diabetes [19], [36], [37]. Recently, Oh et al. [35] reported an increase in CAV1 expression in skeletal muscle cells upon 17-β-estradiol (E2) treatment, whereas dihydrotestosterone (DHT) had no effect.

A wealth of information exists concerning the functional aspects of CAV1, including its active site for downstream signaling molecules as well as roles in lipid transport, TG transport, and nutrient storage [38]–[40]. Recently, it has been reported that CAV1 is an important target protein in E2- as well as DHT-dependent regulation of various metabolic pathways, particularly in cancer and diabetes [33], [35], [41], [42].

Previously, we observed increased expression of CAV1 in adipose tissues of obesity- resistant SD male rats fed a HFD [43]. In this sense, we can hypothesize that diet affects the expression of CAV1 in a sex and site-specific manner. Moreover, we also postulate that CAV1 may serve as an important player affecting sex dimorphism in the development of obesity and other metabolic diseases.

To date, no report is available concerning the sex-dimorphic regulation of CAV1 in response to diet and/or sex hormone treatment. Therefore, the main objective of this study was to elucidate the molecular mechanism underlying the effects of sex steroid hormones on sex-dimorphic regulation of CAV1, which is a potent target protein in the treatment of lipodystrophy and other complications resulting from altered hormonal regulation of adipocyte metabolism. To this end, we applied the major physiological sex hormones E2 and DHT *in vivo* (Sprague-Dawley rats) as well as *in vitro* (3T3-L1 and HIB1B cell lines) to elucidate whether or not both sex hormones actually regulate CAV1 expression levels in various adipose tissues. In addition, CAV1 knockdown (KD) using a siRNA transfection system was performed to determine the effects of CAV1 on metabolic as well as sex hormone receptor candidates in both adipocyte cells. Lastly, using co-immunoprecipitation, we identified previously unrecognized CAV1-interacting partner proteins in adipose tissues, including members of mitochondrial or lipid pathways.

## Materials and Methods

### Ethics Statement

All animal experiments were approved by the Committee for Laboratory Animal Care and Use of Daegu University. All procedures were conducted in accordance with the Guide for the Care and Use of Laboratory Animals published by the National Institutes of Health.

### Animals, Diets, and Hormone Treatments

Five-week-old Sprague-Dawley (SD) rats were purchased from Daehan Experimental Animals (Seoul, Korea) and maintained under control conditions: temperature (23±2°C), humidity (55%) in a controlled room, normal chow and tap water, and a 12 h light/12 h dark cycle for 1 week for acclimatization. We treated SD rats with 17-β-estradiol (E2) and dihydrotestosterone (DHT) without ovariectomy (OVX) or orchiectomy (ORX) in order to verify the effects of external sex hormones without the adjacent physiological consequences of OVX or ORX. To eradicate the initial difference in endogenous sex hormone concentration between the groups, we measured plasma sex hormone concentrations before starting the animal experiment. At the beginning of the experiment, animals having at least 2 times higher or lower hormone levels compared to the rest of the animals were excluded. Average sex hormone values were as follows; E2 concentration in males was 0.33 pg/ml, E2 concentration in females was 0.34 pg/ml and DHT concentration in males was 1.87 ng/ml, DHT concentration in females was 1.77 ng/ml. Remaining rats with similar sex hormone concentrations were grouped randomly into six rats/group. Later, male and female rats were divided into six groups each containing six rats selected randomly, resulting in total 12 groups viz; male and female controls fed a normal diet (ND CON), E2-treated males and females fed a normal diet (ND E2), DHT-treated males and females fed a normal diet (ND DHT), male and female controls fed a high fat diet (HFD CON), E2-treated males and females fed a high fat diet (HFD E2), and DHT-treated males and females fed a high fat diet (HFD DHT). All animals were fed either ND or HFD with or without either E2 (Sigma, St. Louis, MO) or DHT (Sigma) treatment. Sex hormones were first dissolved in ethanol and diluted in 0.15 M NaCl solution and then injected intraperitoneally to a final concentration of 750 µg of sex hormone/kg of body weight or the same volume of vehicle for 48 days at 3 day intervals. ND and HFD contained 12% and 45% fat as an energy source (Korea Lab., Hanam, Korea), and the dietary composition was the same as that used in our previous study [29]. All rats and food were weighed every week for 6 weeks. All animals were fasted for 10–12 hrs (overnight) and were sacrificed using cervical dislocation.

### Blood Samples and Plasma Biochemical Characterization

Blood samples were collected by puncturing the tail vein or abdominal aorta and collected into EDTA-tubes (BD, Franklin Lakes, NJ, USA). Plasma was separated by centrifugation (3,000×*g*, 10 min), followed by storage at −80°C until further analysis. Common plasma biochemical characterizations were performed using a commercial ELISA kit for plasma TG, FFA (Biovision Inc. Milpitas, CA, USA), E2 (Cayman Chemical Company, Ann Arbor, MI, USA), and DHT (Bioassay Technology Laboratory, Shanghai, Yangpu, China). All ELISA experiments were carried out in triplicate using individual plasma samples.

### Collection of Tissues and Protein Sample Preparation

Three different rat WATs (inguinal, abdominal, and gonadal WATs) interscapular BAT, liver and S Muscle were collected immediately after sacrifice, weighed, washed in saline water, and pulverized in liquid nitrogen, followed by final storage in −80°C. From each WAT and BAT sample, one small portion was stored in 4% *p*-formaldehyde for histological study. Tissue lysates were prepared with RIPA buffer (Sigma-Aldrich), homogenized, and centrifuged at 12,000×*g* for 20 min. For immunoblot analysis, protein samples were prepared from six individual tissue samples of each group, followed by quantification and polling of six protein samples to make a single sample from each group.

### Immunoblot Analysis

The lysates were mixed with 5X sample buffer (50 mM Tris of pH 6.8, 2% SDS, 10% glycerol, 5% β-mercaptoethanol, and 0.1% bromophenol blue) and heated for 5 min at 95°C, followed by SDS-polyacrylamide gel electrophoresis (PAGE) using 8, 10, or 12% (w/v) polyacrylamide gel. After electrophoresis, samples were transferred to a polyvinylidene difluoride membrane (PVDF, Roche Diagnostics, Indianapolis, IN, USA) and then blocked for 1 h with TBS (Tris-buffered saline)-T buffer (10 mM Tris-HCl, 150 mM NaCl, and 0.1% Tween 20 containing 5% skim milk). The membrane was rinsed three times consecutively with TBS-T buffer, followed by incubation for 1 h with 1∶1000 dilutions of primary monoclonal anti-β-actin, anti-PPARγ, anti-AMPKα1, anti-HSL, anti-AR, anti-ERα, anti-ERβ, anti-CYP19, anti-UCP1 (Santa Cruz Biotechnology, Santa Cruz, CA, USA), anti-FAS, and anti-CAV1 (Cell Signaling Technology, Beverly, MA, USA) antibodies in TBS-T buffer containing 1% skim milk. After three washes, the membrane was incubated for 1 h with horseradish peroxidase-conjugated anti-goat/rabbit/mouse IgG secondary antibody (1∶1000, AB Frontier, Seoul, Korea) in TBS-T buffer containing 1% skim milk. Development was carried out using enhanced chemiluminescence (Westzol, iNtRON Biotechnology, Seongnam, Korea). Band intensities were normalized using β-actin bands in each tissue (Figure S2) and quantified using ImageMaster (GE Healthcare, Little Chalfont, Buckinghamshire, UK).

### Co-immunoprecipitation (CO-IP) and Protein Mass Fingerprinting (PMF)

Co-immunoprecipitation to identify CAV1-interacting proteins from abdominal WAT and BAT were performed using standard protocol as specified by the CO-IP kit manufacturer (Thermo Scientific, Waltham, MA, USA) and employing anti-CAV1 antibody (Cell Signaling). For the positive control sample, control rabbit IgG (Bethyl Laboratories Inc., Montgomery, TX, USA) was used. After CO-IP, all samples were separated by SDS-PAGE followed by silver staining. Lastly, to identify proteins by peptide mass fingerprinting, protein bands were excised, digested with trypsin (Promega, Madison, WI), mixed with α-cyano-4-hydroxycinnamic acid in 50% acetonitrile/0.1% TFA, and subjected to MALDI-TOF analysis (Microflex LRF 20, Bruker Daltonics, Billerica, MA, USA). Peak list was generated using Flex Analysis 3.0 followed by protein identification using MASCOT, the Matixscience (http://www.matrixscience.com).

### Cell Culture and Differentiation

3T3-L1and HIB1B preadipocytes were cultured and differentiated using a previously used protocol [44], [45]. 3T3-L1 was purchased from Korean Cell line Bank (KCLB10092.1) and HIB1B cell line was a kind gift from Dr. Kwang-Hee Bae [46]. Sex hormone stocks were made with ethanol and later diluted using phosphate buffered saline (PBS) before treatment. During treatment, cells were maintained in maturation medium containing 0–20 µM/ml of E2 (Sigma) or DHT (Sigma) or vehicle (PBS) for 6 days, and maturation medium was changed every 2 days.

### Immunofluorescence

3T3-L1 and HIB1B cells were cultured on sterilized coverslips placed in 6-well plates following the treatment protocol described in the cell culture section. After maturation for 6 days, the cells were washed with PBS and fixed with 10% formaldehyde. To measure the immunofluorescence of abdominal WAT and BAT, 4% *p*-formaldehyde-fixed and paraffin- embedded tissues were processed by standard protocol followed by antigen retrieval using 10 mM sodium citrate solution. Fixed adipocytes or tissue sections were washed with PBS-Tween 20 three times and blocked with 5% BSA for 1 h. Cells were then incubated with anti-CAV1 antibody (1∶200 dilution) overnight in 4°C, washed three times with PBS-T, and incubated with FITC-conjugated secondary antibody for 1 h, after which the coverslip was mounted onto a slide with DAPI-containing mounting medium (Invitrogen, Carlsbad, CA, USA). Fluorescence images were taken by an Olympus IX51 inverted microscope (Olympus Co., Shinjuku, Tokyo, Japan). Abdominal adipocyte area and BAT lipid area were quantified using Image J software and compared within groups to check hormonal effect on adipocyte morphology.

### Oil Red O Staining and Quantification of Triglycerides (TG)

All cell types were matured for 6 days, washed with phosphate-buffered saline (PBS), fixed with 10% formalin for 1 h at room temperature, and washed three times with deionized water. A mixture of Oil Red O (0.6% Oil Red O dye in isopropanol) and water at a 6∶4 ratio was layered onto the cells for 10 min, followed by washing three times with deionized water and photography. The matured cells were washed twice with PBS and harvested in order to prepare cell lysate using RIPA buffer (Sigma). TG content was measured according to the manufacturer’s instructions using a TG test kit (Asan Pharm. Co., Yeongcheon, Korea). Absorbance was measured at 550 nm, and TG content was normalized to protein content as determined by the Bradford method [47].

### Quantitative Real-time RT-PCR

Total RNA was isolated using a total RNA isolation kit (RNA-spin, iNtRON Biotechnology, Seongnam, Korea) from each group of cells matured for 4–6 days in corresponding maturation medium, after which 1 µg of RNA was converted to cDNA using Maxime RT premix (iNtRON Biotechnology). Transcript levels of genes were quantitatively determined by employing Power SYBR Green (GE Healthcare, Warrington, UK) with real-time RT-PCR (Stratagene 246 mx 3000p QPCR System, Agilent Technologies, Santa Clara, CA, USA). Transcript levels of each gene were normalized to the level of *β-actin*. Sequences of primer sets used in this study are listed in Table 1.

**Table 1:** Sequences of primers for real-time RT-PCR used in this study.

Genes		Primer sequence (5′ - 3′)
*Cav1*	F^a)^	ACCTCTCTGGACTGGCAGAA
	R^b)^	TCCCTGGAGGTTCACTCATC
*Pparγ*	F	GGTGAAACTCTGGAGATTC
	R	CAACCATTGGGTCAGCTCTT
*C/ebpα*	F	AGGTGCTGGAGTTGACCAGT
	R	CAGCCTAGAGATCCAGCGAC
*Pparα*	F	CCCCACCAGTACAGATGAGTC
	R	GGAGTTTTGGGAAGAGAAAGG
*Me1*	F	AGAGCTCTCGTTCCCAAACA
	R	TTGTCTCAGAGGTGGGTTCC
*Fasn*	F	CCTTAGAGGCAGTGCAGGAC
	R	TTGCTGCACTTCTTGGACAC
*Fabp4*	F	CACCTGGAAGACAGCTCCTC
	R	AATCCCCATTTACGCTGATG
*Prkaa1*	F	CAGGCCATAAAGTGCAGTTA
	R	AAAAGTCTGTCGGAGTGCTGA
*6Pgd*	F	TGAAGGGTCCTAAGGTGGTCC
	R	CCGCCATAATTGAGGGTCCAG
*Srebp-1C*	F	ACTGTCTTGGTTGTTGATGAGCTGGA
	R	ATCGGCGCGGAAGCTGTCGGGGTAG
*Esr1*	F	AAAGGGATTCCAGGGCTAAA
	R	CGCTTTGTCAACGACTTCAA
*Esr2*	F	GAAGCTGGCTGACAAGGAAC
	R	AACGAGGTCTGGAGCAAAGA
*Ar*	F	GGACCATGTTTTACCCATCG
	R	TCGTTTCTGCTGGCACATAG
**Internal control**		
*β-Actin*	F	AGCCATGTACGTAGCCATCC
	R	CTCTCAGCTGTGGTGGTGAA

F^a)^, sequence from sense strands; R^b)^, sequence from anti-sense strands.

### Knockdown of Cav1

A commercially available siRNA transfection system (Santa Cruz Biotechnology), which is specific to *Cav1* (a pool of three target-specific 20–25 nucleotide siRNAs designed to knockdown gene expression) was used for gene silencing in both 3T3-L1 and HIB1B cells. Post-confluent cells in 6-well culture dishes were washed twice with transfection medium and overlaid with a previously made mixture of siRNA and transfection reagent (Santa Cruz Biotechnology). The transfection process was carried out for 5–7 h depending on cell condition, after which cells were maintained in maturation medium for 6–8 days with regular medium changes every 2 days. Transfection efficiency was monitored by estimating the uptake of non-targeting fluorescein-labeled double-stranded RNA oligomers (BLOCK-iT, 150 nM per well, #2013, Invitrogen). Transcript levels of each gene were normalized to the level of *β-actin*. Finally, knockdown efficiency of transiently siRNA-expressing cells was determined as follows:
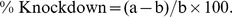



a: Normalized expression level of each gene in siRNA-transfected cells.

b: Normalized expression level of each gene in BLOCK-iT-transfected cells.

### Statistical Analysis

Statistical significance between control and hormone treated groups were compared using Student’s *t-*test. Group means were considered significantly different at *p*<0.05. The significance of the effects of sex, diet and hormonal treatments was tested using multivariate ANOVA (M-ANOVA). All of the statistical tests were performed using SPSS 17.0 software (Technologies, Chicago, IL, USA). The level of significance was set at *p*<0.05.

## Results

### E2 and DHT Oppositely Affect Body Weight Gain and Fat Mass in SD Rats

**Figure 1:**
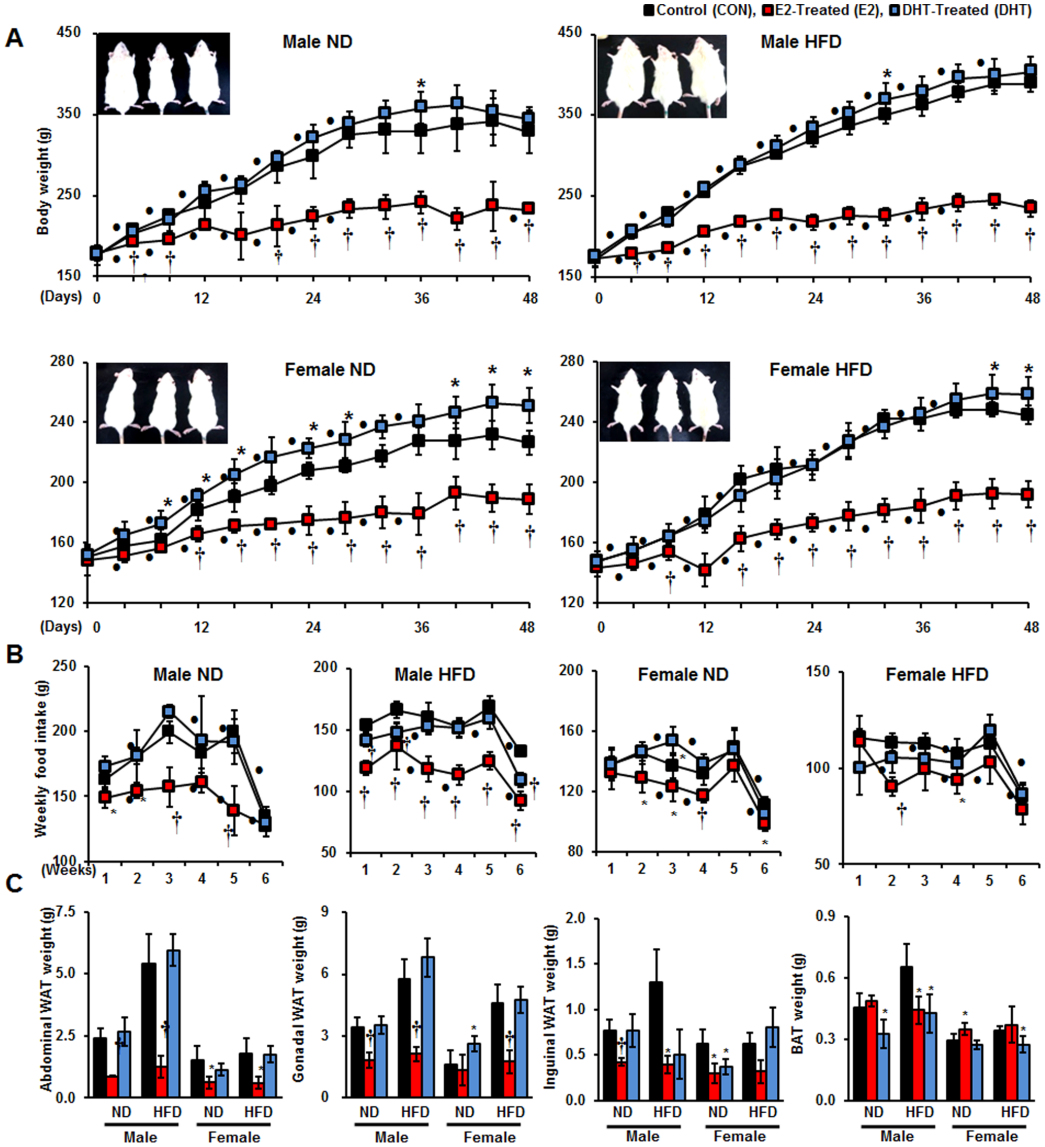
Dietary and hormonal effects on time-dependent body weight gain with whole body images (inside panels: control, E2-treated, and DHT-treated rats from left) of each group (A), weekly food intake (B), and weights of different adipose tissue depots (C). Statistical significance between control and hormone treated groups were calculated by Student’s *t-*test, where, *p<0.05 and †*p*<0.01. Significances between time dependent body weight gain and weekly food intake were calculated using repeated measures multivariate ANOVA, where • represents *p*<0.05.

**Figure 2:**
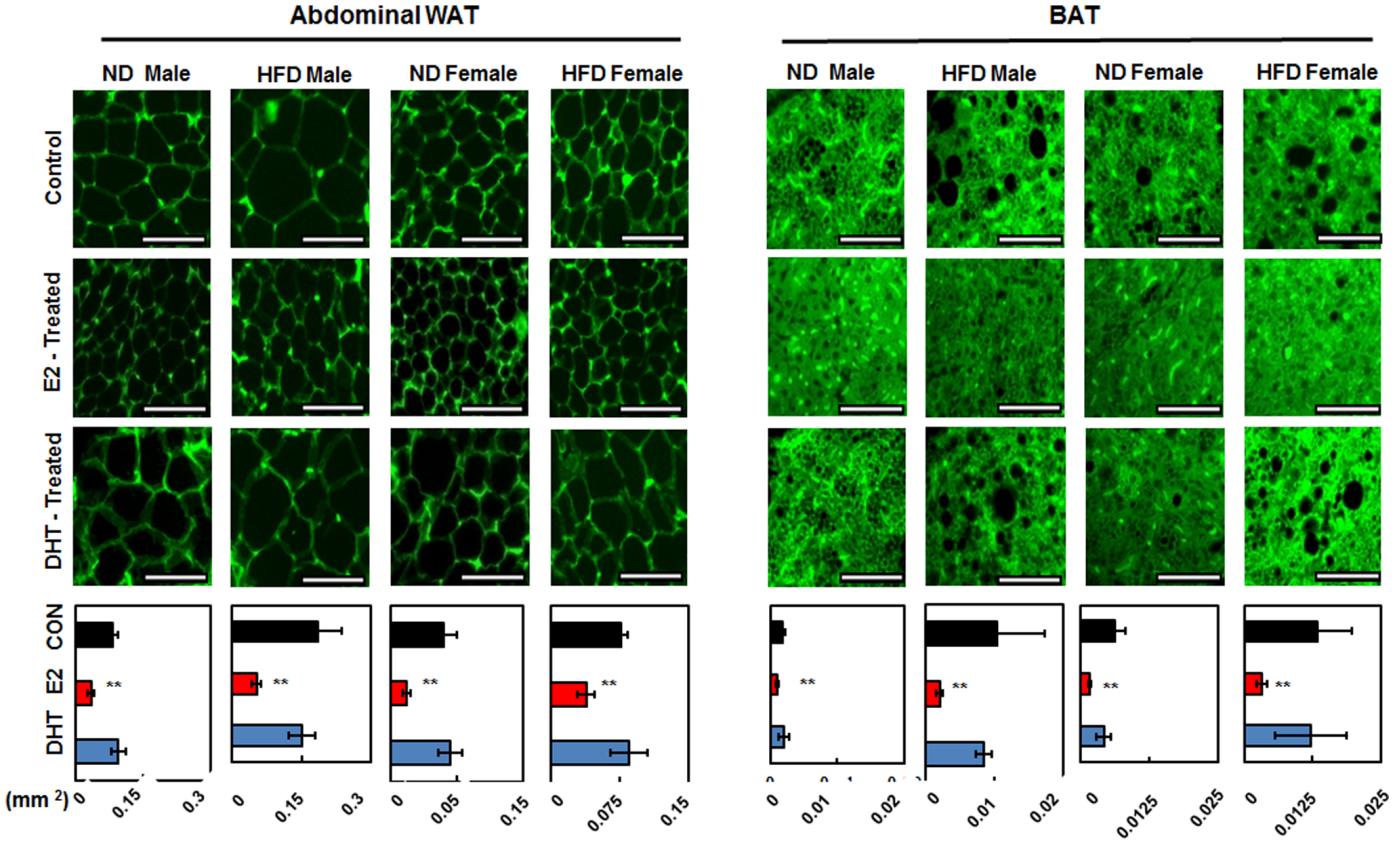
Dietary and hormonal effects on morphologies of abdominal WAT and BAT using immunofluorescence of CAV1-FITC. Scale bar represents 100 µM and 50 µM, respectively. Adipocyte area or lipid area between control and hormone treated groups were calculated by Student’s *t-*test, where, *p<0.05 and **p<0.01. The significance of the effects of sex, diet and hormonal treatments was tested using multivariate ANOVA (M-ANOVA), where *p*<0.05.

E2 and DHT treatments to 5-week-old SD rats for 48 days showed opposite effects on body weight gain in all groups regardless of sex or diet. E2-treated rats exhibited greatly reduced body weight gain during the entire time period starting from the 4^th^ day in a time-dependent manner (Figure 1A). Conversely, DHT showed stimulatory effects on body weight gain, although its effect was not as prominent as E2 in all groups (Figure 1A). The most significant body weight increase in response to DHT treatment was detected in the ND female group. Food consumption of each group was monitored weekly, as shown in Figure 1B. E2 treatment led to a marked reduction of food intake in all groups, whereas DHT-treated rats showed no significant difference of food intake (Figure 1B). Finally, collected WAT showed significant attenuation of fat pad weight in E2-treated groups (Figure 1C). On the contrary, DHT-treated groups showed only slight differences from control counterparts. Interestingly, the effect was reversed for BAT, as E2 treatment elevated BAT fat pad weight especially in females while DHT reduced BAT weight in all groups, *albeit* not significantly (Figure 1C; Table S1). As abdominal WAT and BAT showed the most interesting fat pad weight patterns, we studied the histological characteristics of these tissues by immunoflourescence using CAV1-FITC followed by quantification of adipocyte area and lipid area in abdominal WAT and BAT, respectively. Expectedly, E2-treated abdominal WAT exhibited smaller adipocyte size than those of the other two groups (Figure 2; Table S2). BAT also showed a similar pattern with nearly invisible lipid droplets in E2-treated groups. We also found that plasma biochemical parameters reflected attenuation of TG in all E2-treated groups except female groups fed ND (Figure S1). In addition, plasma FFA levels significantly decreased in HFD-fed males but increased in HFD-fed females. In E2-treated groups, plasma E2 concentrations were significantly higher in all groups except HFD females. In contrast, plasma DHT levels showed no significant change in DHT-treated HFD rats, whereas DHT levels increased in E2-treated HFD rats (Figure S1).

### E2 and DHT Oppositely Regulate CAV1 in Different Adipose Tissue Depots

**Figure 3:**
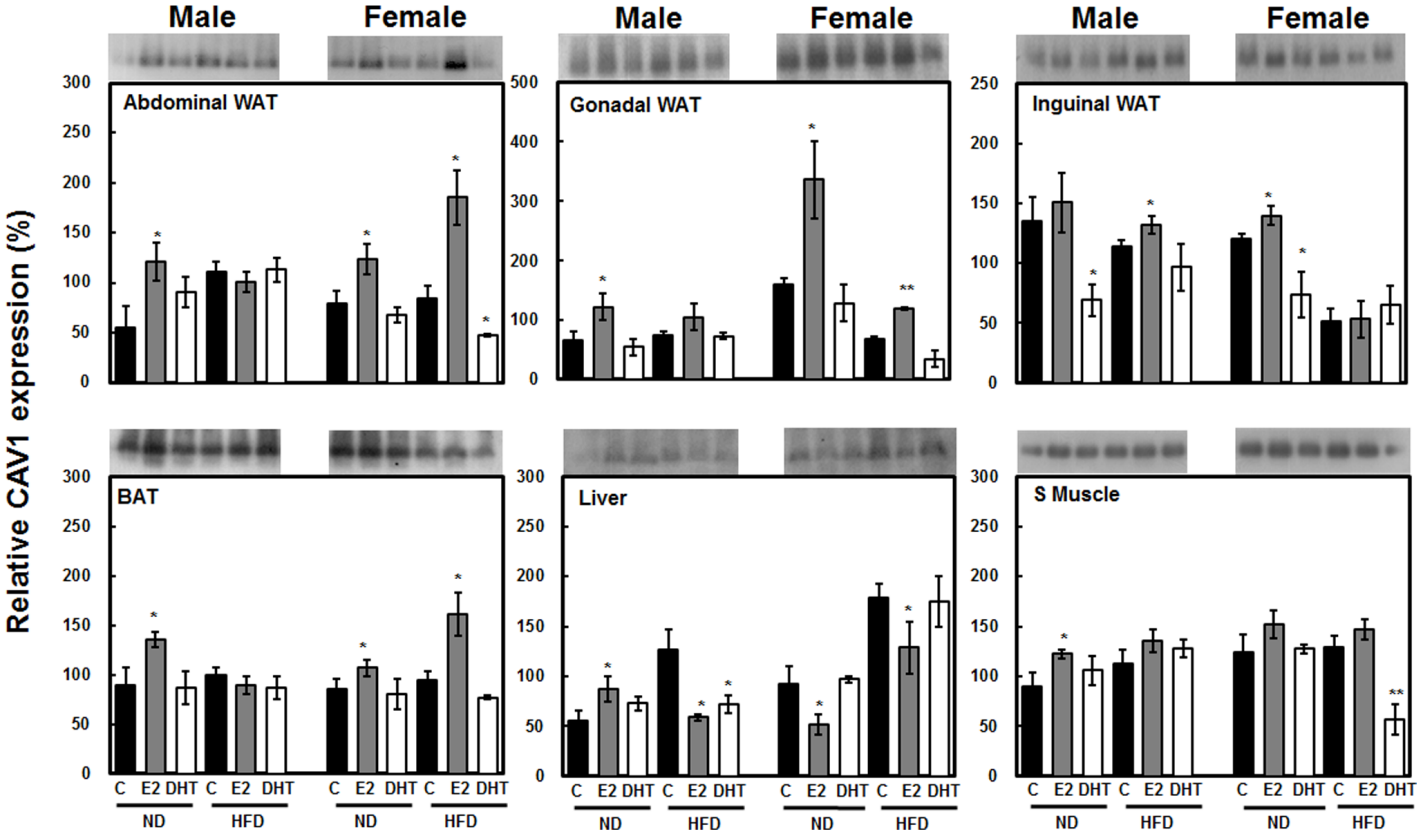
CAV1 was differentially expressed in tissues upon treatments with high fat diet and sex steroid hormones (E2 and DHT) in a sex-dependent manner. Band densities were normalized using β-actin in each tissue to calculate relative intensity (%). Statistical significance between control and hormone treated groups were calculated by Student’s *t-*test, where, *p<0.05 and **p<0.01. The significance of the effects of sex, diet and hormonal treatments was tested using multivariate ANOVA (M-ANOVA), where *p*<0.05.

To elucidate the regulatory patterns of CAV1 after sex hormone treatment, we measured CAV1 expression in three different adipose tissue depots by immunoblot analysis. Interestingly, E2 treatment resulted in elevated levels of CAV1 in WAT of most groups (Figure 3). Most prominently, marked stimulation of CAV1 was observed in gonadal WAT of both sexes and diet groups (Figure 3). In contrast, CAV1 expression was not significantly reduced in either abdominal or gonadal WAT by DHT treatment, except in HFD-fed female groups (Figure 3). In BAT, E2 significantly stimulated CAV1 expression especially in females (Figure 3). To our surprise, abdominal WAT and BAT showed similar CAV1 expression levels with no significant difference in the HFD male group (Figure 3). Lastly, we measured CAV1 expression in two other major metabolic target tissues viz. the liver and soleus skeletal muscle (S muscle). E2 treatment caused significant reduction of CAV1 expression in the liver, especially in females (Figure 3). In contrast, DHT treatment did not have significant effects (Figure 3). Both E2 and DHT applied to S muscle had no marked effects on CAV1 expression, except in HFD-fed females, whereas DHT treatment significantly reduced CAV1 expression (Figure 3). Taken together, CAV1 was regulated in a tissue-specific manner with greater sex hormone dependence in adipose tissues and the liver (Figure 3).

### Sex Hormones Regulate Hormone Sensitive Lipase (HSL) in WAT and Uncoupling Protein 1 (UCP1) in BAT

**Figure 4:**
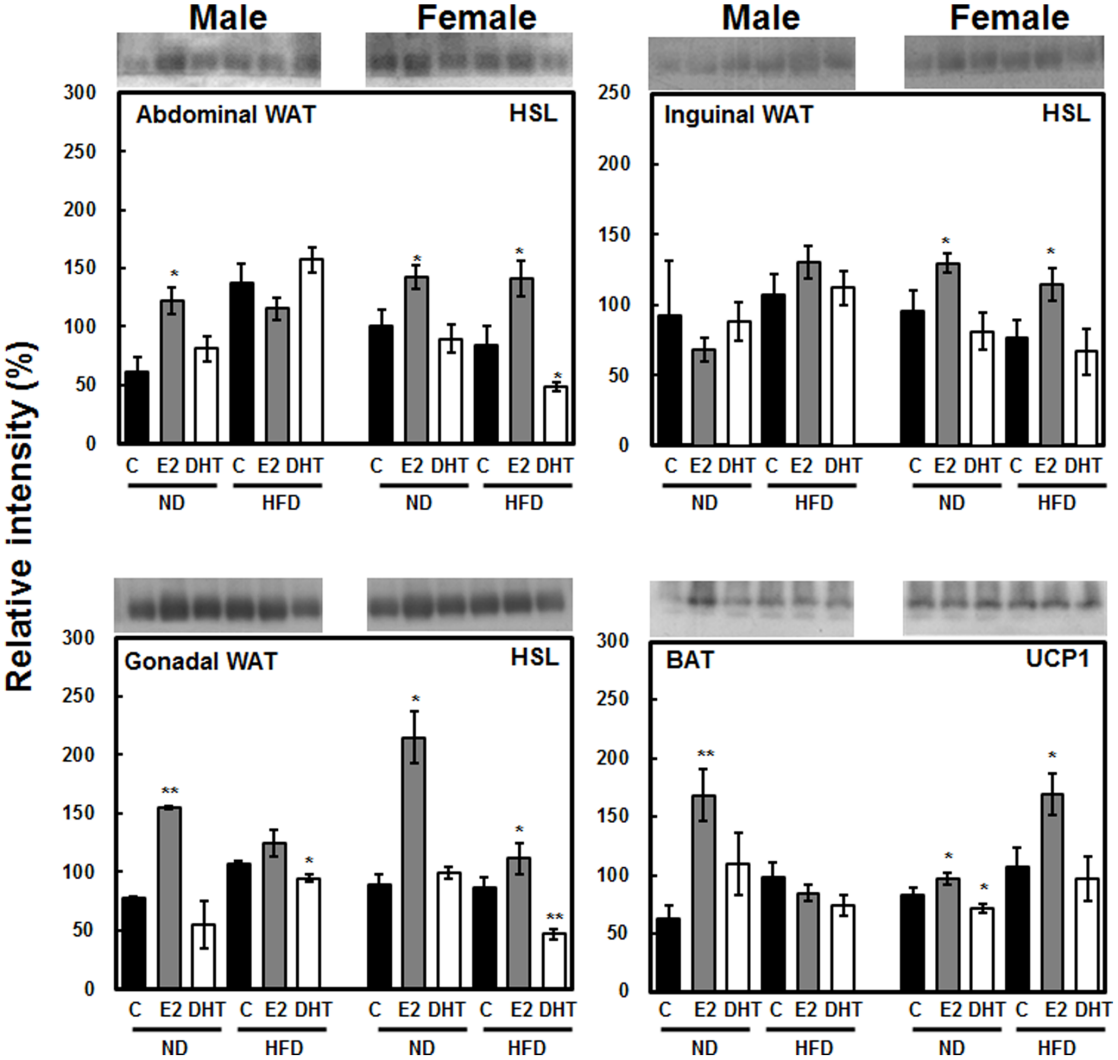
Differential expression of hormone sensitive lipase (HSL) in WAT and uncoupling protein 1 (UCP1) in BAT before and after treatments with high fat diet and sex steroid hormones in both sexes. Band densities were normalized using β-actin in each tissue to calculate relative intensity (%). Statistical significance between control and hormone treated groups were calculated by Student’s *t-*test, where, *p<0.05 and **p<0.01. The significance of the effects of sex, diet and hormonal treatments was tested using multivariate ANOVA (M-ANOVA), where *p*<0.05.

In order to investigate the existence of sex dimorphism in lipolysis and thermogenesis in response to diet and sex hormone treatments, expression levels of HSL and UCP1 were determined in different WAT and BAT depots. As expected, E2 greatly stimulated HSL expression in WAT along with UCP1 expression in BAT in most groups, suggesting higher lipolytic and thermogenic capacity in E2-treated rats, as well as more prominent stimulation in female groups (Figure 4). On the contrary, DHT-treated females showed either unchanged or attenuated expression patterns of HSL and UCP1. Similar to the expression pattern of CAV1 in abdominal WAT and BAT, neither HSL nor UCP1 were significantly altered in the HFD male group, which may explain why this group underwent the highest weight gain (Figure 4).

### Differential Expression of Sex Hormone Receptors and Aromatase in Response to E2 and DHT Treatments

**Figure 5:**
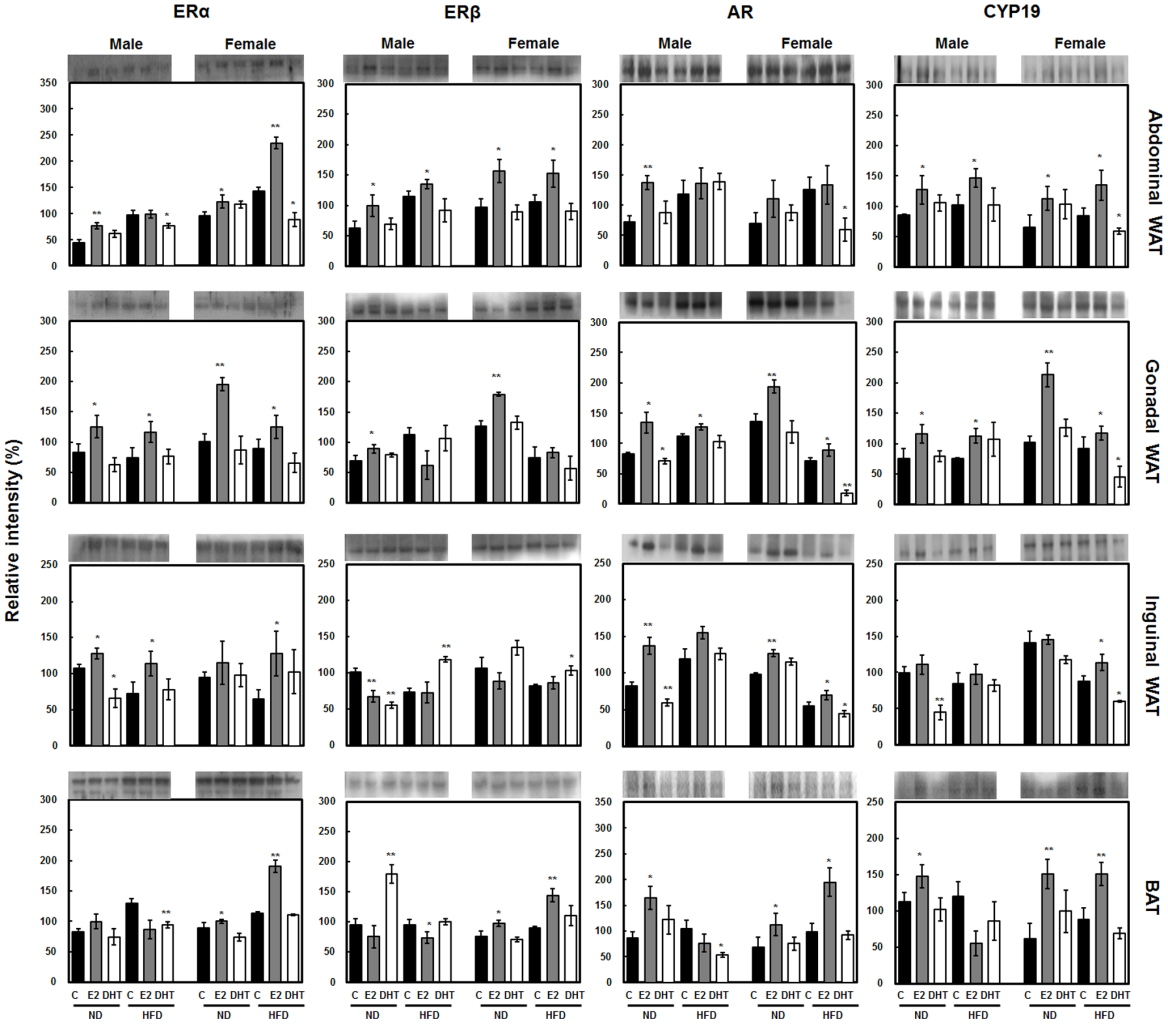
Sex-dimorphic differential expression of estrogen receptors (ERα and ERβ), androgen receptor (AR), and aromatase (CYP19) in different adipose tissue depots. Band densities were normalized using β-actin in each tissue to calculate relative intensity (%). Statistical significance between control and hormone treated groups were calculated by Student’s *t-*test, where, *p<0.05 and **p<0.01. The significance of the effects of sex, diet and hormonal treatments was tested using multivariate ANOVA (M-ANOVA), where *p*<0.05.

In order to examine whether or not sex hormone receptors and aromatase are important targets in the regulation of CAV1, we determined the levels of estrogen receptors (ERα and ERβ), androgen receptor (AR), and aromatase (CYP19) in various WAT and BAT depots. After E2 treatment, ERα levels were significantly elevated in most adipose tissue depots, most significantly in gonadal WAT where ERα was stimulated in all groups (Figure 5). DHT treatment reduced expression of ERα only in abdominal WAT and BAT of HFD-fed groups (Figure 5). ERβ expression in abdominal WAT was also positively regulated in E2-treated groups with a more prominent effect in females (Figure 5). ERβ expression was stimulated in gonadal WAT and BAT of females but reduced in BAT and gonadal WAT of HFD-fed males (Figure 5). In inguinal WAT, ERβ expression showed no consistent pattern with higher expression in DHT-treated HFD groups (Figure 5). AR showed a differential expression pattern in inguinal WAT, gonadal WAT, and BAT, as it was significantly elevated in E2-treated groups as well as reduced in gonadal and inguinal WAT of DHT-treated ND male and HFD female groups (Figure 5). Further, AR expression significantly increased only in abdominal WAT of ND male groups and decreased in HFD females (Figure 5). Finally, we observed that CYP19 expression was enhanced in abdominal and gonadal WAT by E2 treatment. On the other hand, DHT treatment had a significant effect only in HFD females displaying reduced CYP19 expression. A prominent increase in CYP19 expression was observed in BAT of all E2-treated groups except male HFD groups. Interestingly, DHT treatment attenuated expression of CYP19 in inguinal WAT, but the effect was only significant in ND male and HFD female groups.

### CAV1 Interacts with Major Metabolic as well as Mitochondrial Proteins

**Figure 6:**
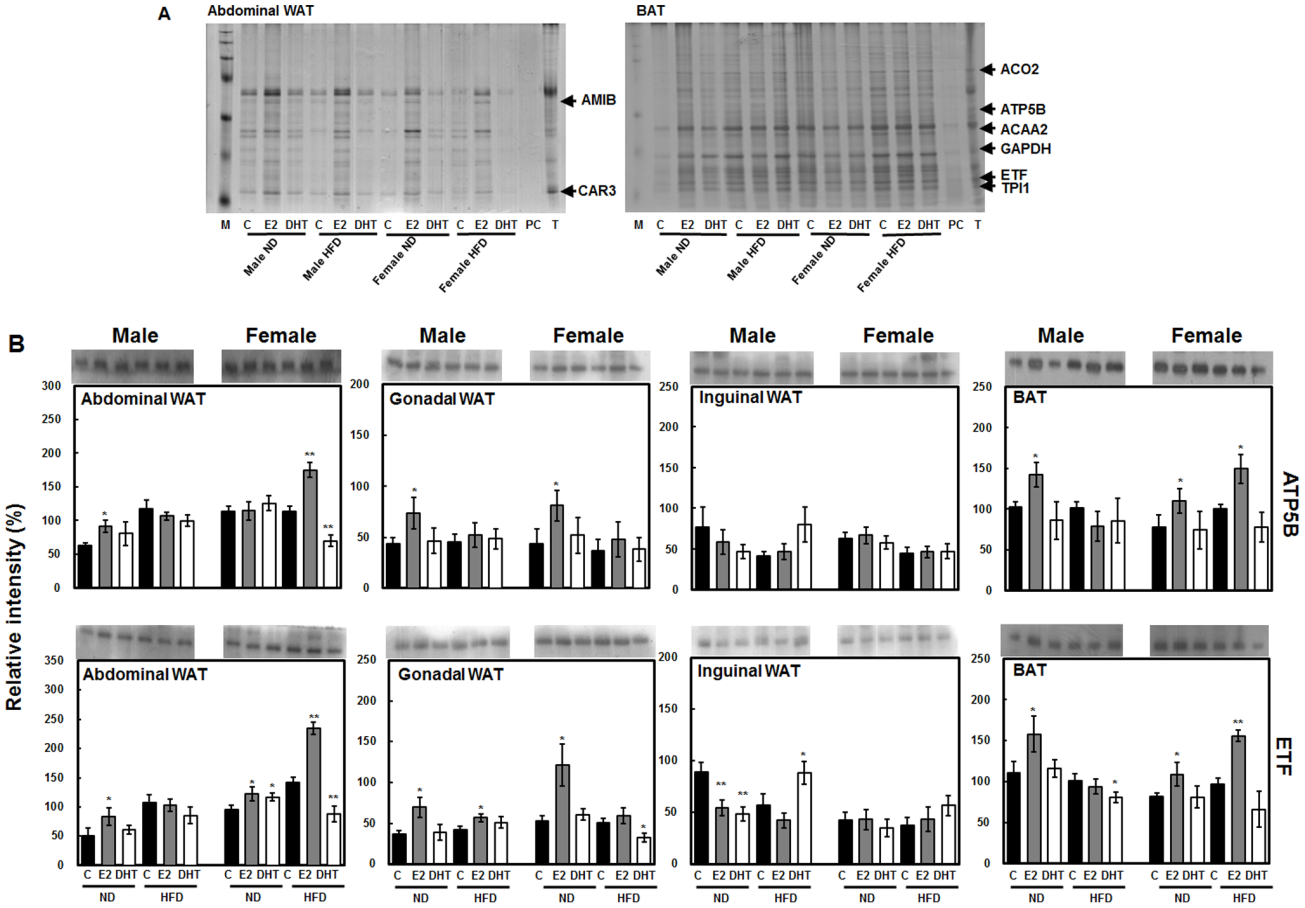
Representative silver-stained SDS-PAGE images of co-immunoprecipitated samples of abdominal WAT and BAT (M: maker, C: control, E2: E2-treated, DHT: DHT-treated, PC: positive control, T: total protein). Proteins were identified by PMF and indicated by black arrows along with their abbreviated names. AMIB: *N*-acetylmuramoyl-*L*-alanine amidase; CAR3: carbonic anhydrase 3; ACO2: aconitate hydratase, mitochondrial precursor; ATP5B: ATP synthase (H^+^ transporting, mitochondrial F1 complex, beta polypeptide); ACAA2: acetyl-CoA acyltransferase 2, mitochondrial; GAPDH: glycerol 3-phosphate dehydrogenase; ETF: electron transfer flavoprotein, subunit beta (ETF); TPI1: triose phosphate isomerase 1 (A). Protein levels of two major mitochondrial proteins, ETF and ATP5B, were identified by PMF in abdominal WAT and BAT (B). Band densities were normalized using β-actin in each tissue to calculate relative intensity (%). Statistical significance between control and hormone treated groups were calculated by Student’s *t*-test, where, *p<0.05 and **p<0.01. The significance of the effects of sex, diet and hormonal treatments was tested using multivariate ANOVA (M-ANOVA), where *p*<0.05.

To identify novel CAV1 partner proteins that may play roles in controlling the metabolic effects of sex hormones, we performed co-immunoprecipitation (Co-IP) of CAV1-associated proteins followed by SDS-PAGE and protein mass fingerprinting (PMF). Co-IP experiments were performed using abdominal WAT and BAT, as these two depots are considered to be the most important depots in sex-differential metabolic diseases. PMF data identified several major metabolic interacting proteins, as shown in Figure 6A. In abdominal WAT, we managed to identify two proteins viz. *N*-acetylmuramoyl-*L*-alanine amidase (AMIB) and carbonic anhydrase 3 (CAR3). In the case of BAT, we identified six interacting proteins, including aconitate hydratase, mitochondrial precursor (ACO2), ATP synthase (H^+^ transporting, mitochondrial F1 complex, beta polypeptide, ATP5B), acetyl-CoA acyltransferase 2, mitochondrial (ACAA2), glyceroldehyde-3-phosphate dehydrogenase (GAPDH), electron transfer flavoprotein, subunit beta (ETF), and triose phosphate isomerase 1 (TPI1) (Figure 6A). A majority of the identified proteins in BAT are mitochondrial proteins, with ATP5B and ETF being electron transport family members involved in energy expenditure. We reconfirmed the expression levels of ATP5B and ETF in all adipose tissue depots by immunoblot analysis. The two proteins were the most prominently regulated in BAT, and E2 treatment enhanced protein expression in all groups except HFD-fed males (Figure 6B). Most interestingly, this expression pattern was the same as that of CAV1, which indicates that ATP5B and ETF are highly associated with CAV1. Moreover, ETF showed the same expression pattern as CAV1 in abdominal WAT, whereas E2 treatment markedly affected the expression of ATP5B specifically in ND-fed males and HFD-fed females (Figure 6B). In the case of gonadal WAT, E2 stimulated ETF expression in all groups, whereas ATP5B was stimulated only in ND groups of both sexes. Inguinal WAT showed no significant differences in expression between these two proteins, especially in HFD groups (Figure 6B).

### E2 and DHT Oppositely Regulate CAV1 Expression in 3T3-L1 and HIB1B Adipocytes

**Figure 7:**
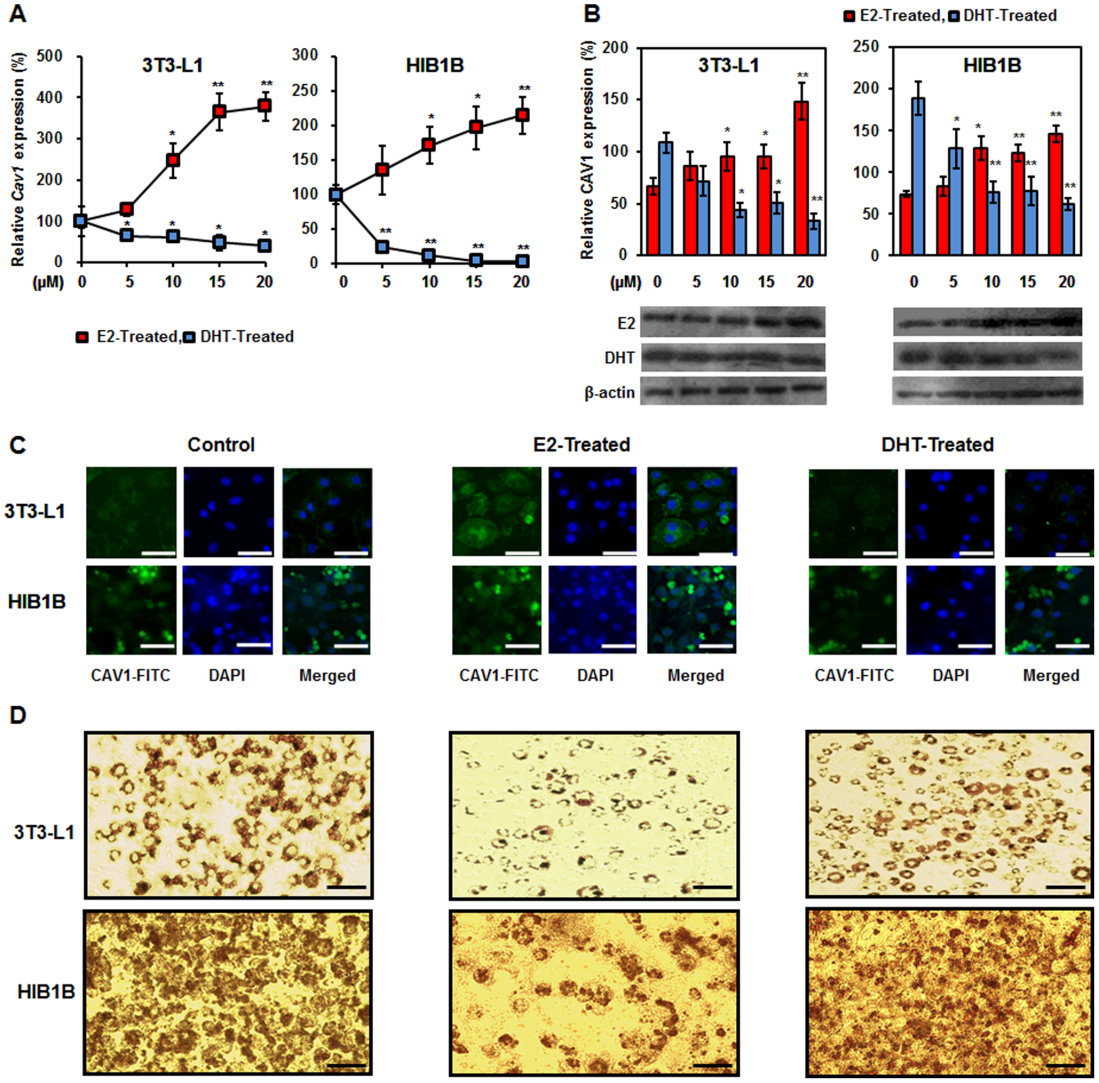
Effects of sex hormone treatments on *Cav1* expression in 3T3-L1 and HIB1B adipocytes (A), CAV1 protein expression levels (B), CAV1 expression levels by immunocytochemistry (C), and fat accumulation as determined by Oil Red O staining (D). Scale bar represents 50 µM and 25 µM, respectively. Statistical significance between different groups was calculated by Student’s *t*-test, where *p<0.05 and **p<0.01.

To validate data obtained from *in vivo* experiments, we conducted a series of *in vitro* experiments using 3T3-L1 and HIB1B cell lines, which are widely used for biological research on adipose tissues (WAT and BAT, respectively). Caveolin 1 mRNA and protein expression levels significantly increased in a dose-dependent manner after treatment with 0, 5, 10, 15, 20 µM E2 (Figure 7A). Conversely, the same dose of DHT led to the dose-dependent reduction of *Cav1* mRNA expression (Figure 7A). This opposite expression pattern was also observed for CAV1 protein levels (Figure 7B). Since 10 µM E2 or DHT was the lowest concentration showing significant effects on CAV1 expression, all subsequent experiments were carried out at this concentration. In the next step, CAV1 expression levels were evaluated in 3T3-L1 and HIB1B cells by immunofluorescence assay using anti-CAV1 antibody. E2-treated cells showed the highest fluorescence levels, confirming higher expression of CAV1, whereas DHT-treated cells showed diminished fluorescence compared to control cells (Figure 7C).

The effect of E2 or DHT treatment on oil droplet accumulation in 3T3-L1 and HIB1B cells was determined by Oil Red O staining. E2 treatment significantly reduced oil droplet accumulation in both cell types, whereas DHT stimulated oil droplet accumulation (Figure 7D). This result suggests an inverse correlation compared to the CAV1 expression pattern, which led us to hypothesize CAV1 as an anti-adipogenic protein. This result also supports the aforementioned *in vivo* data in which CAV1 expression was positively affected by E2 and negatively affected by DHT, leading to reduced and increased body weight gain, respectively, in rats of both sexes regardless of diet (Figure 1A).

### *Cav1* Knockdown (KD) Stimulates Lipogenesis but Suppresses Sex Hormone Receptors in 3T3-L1 and HIB1B Adipocytes

**Figure 8:**
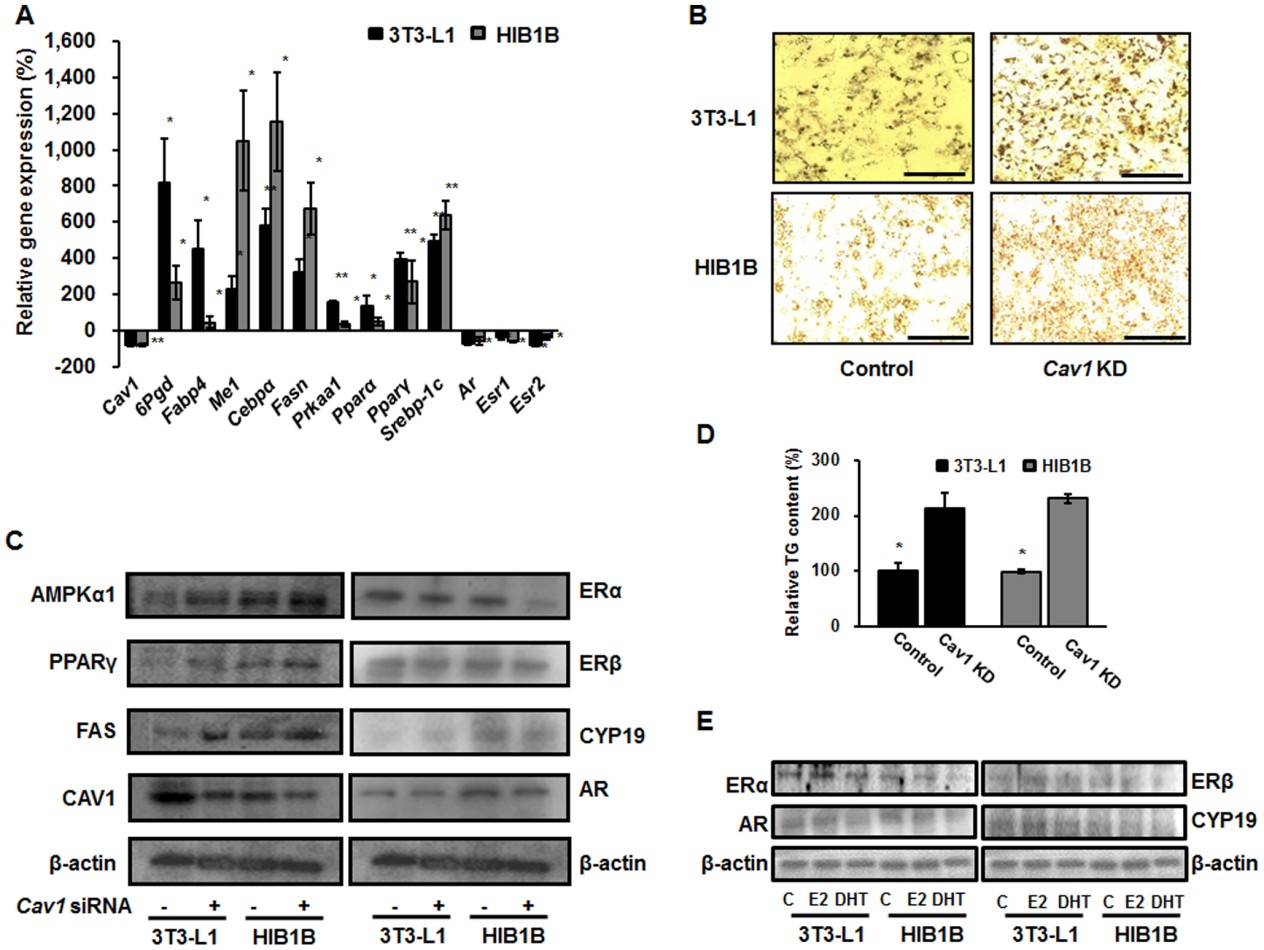
Effect of *Cav1* knockdown (KD) on mRNA expression levels of major metabolic and sex hormone receptors in 3T3-L1 and HIB1B adipocytes (A), fat accumulation (B), protein expression levels of major metabolic and sex hormone receptors (C), triglyceride content (D), as well as protein expression levels of estrogen receptors, androgen receptor, and aromatase before and after sex hormone treatments (E). Scale bar represents 50 µM and 25 µM, respectively. Statistical significance between different groups was calculated by Student’s *t-*test, where *p<0.05 and **p<0.01.

We efficiently knocked down the *Cav1* gene in both cells to demonstrate its effects on total adipogenic capacity and regulation of sex hormone receptors. Surprisingly, when *Cav1* was knocked down, expression levels of representative adipogenic and lipogenic marker genes (e.g. *6Pgd*, *Fabp4*, *Me1*, *Cebpα*, *Fasn*, *Prkaa1*, *Pparα*, *Pparγ*, and *Srebp-1C*) significantly increased (Figure 8A). Protein levels of selected lipogenic proteins were also up-regulated in *Cav1* KD cells (Figure 8C). Furthermore, increases in oil droplet formation and TG concentration were observed in *Cav1* KD cells (Figures 8B, D). On the contrary, *Cav1* KD cells exhibited diminished mRNA and protein expression levels of estrogen and androgen receptors in both cells, reflecting the importance of *Cav1* in sex hormone-dependent regulation of adipocyte metabolism. Importantly, aromatase (CYP19) expression levels were oppositely regulated in 3T3-L1 and HIB1B adipocytes, indicating tissue-specific interconnection between CAV1 and CYP19 (Figure 8C). Additionally, our *in vitro* data showed that E2 and DHT treatments had opposite effects in 3T3-L1 and HIB1B adipocytes. These opposite effects were more prominent on ERα and AR expression, whereas ERβ and CYP19 were stimulated after E2 treatment only in 3T3-L1 cells. However, ERβ expression in HIB1B adipocytes showed attenuated expression upon DHT treatment (Figure 8E).

## Discussion

Based on our previous finding that CAV1 is highly expressed in adipose tissues in obesity-resistant rats fed a HFD [43], we wished to determine exactly which factors are involved in higher CAV1 expression for obesity resistance in HFD-fed rats as well as what different physiological programs exist between male and female rats. In this sense, we hypothesized that CAV1 may act as a potent molecular target for sex hormone-dependent regulation of adipocyte metabolism. The main goal of this study was to elucidate the interconnection between these two intensely studied networks in the context of WAT and BAT metabolism.

The present study demonstrated attenuated body weight gain, WAT weight, and food intake along with increased BAT weight in both E2-treated male and female rats. In contrast, DHT had the opposite effects, which is in line with previous reports in OVX/ORX rodents [22], [48]–[51]. Smaller white adipocytes in WAT and fewer oil droplets in BAT of E2-treated groups were confirmed by CAV1-FITC immunofluorescence imagery, suggesting reduced lipogenic capacity. Additionally, attenuation of BAT weight in DHT-treated rats of both sexes may be connected with the reduced thermogenic capacity in these groups. This is the first report to confirm that exogenous sex hormones without OVX/ORX have opposite effects on body weight parameters in normal SD rats as compared to previous reports [35].

Previous reports have demonstrated the elevated expression of CAV1 after E2 treatment in vascular smooth muscle cells and aortic endothelial cells by transcriptional and translational stimulation [33], [35], [52]. We report here for the first time that CAV1 expression is stimulated by E2 and reduced by DHT in WAT and BAT, as depicted by our *in vivo* and *in vitro* experiments as well as further supported by immunocytochemistry images of adipocytes. These results clearly demonstrate that CAV1 is a novel target protein in the hormonal regulation of adipocyte metabolism. In the current *in vitro* study, we have used supraphysiological concentration of sex hormones firstly to show hormonal regulation of CAV1 and secondly, to connect CAV1 expression levels with corresponding adipogenic capacity of these adipocytes based on the earlier reports [53]–[55], demonstrating beneficial effect of supraphysiological concentration of sex hormones on lipogenesis and lipid metabolism.

We also observed that hormonal regulation of CAV1 is tissue-specific. In adipose tissues and S muscle, E2 showed stimulatory effects on CAV1 expression, whereas DHT had suppressive effects (Figure 3). However, the converse result was observed in the liver, demonstrating that the function of CAV1 varies in different tissues. This idea was first suggested by Cohen et al. [56], who reported that CAV1 is important for insulin signaling in adipose tissue but not in muscle or liver. In addition, Fernandez et al. [57] reported that CAV1 is positively correlated with lipogenesis in the liver. In the current study, E2-treated rats showed reduced lipogenesis associated with lower CAV1 expression (Figure 8). The role of CAV1 in the regulation of liver function is not yet clear. Reduced expression levels of CAV1 in livers of E2-treated male and female rats fed a HFD may indicate reduced lipid accumulation in the liver, as higher expression of CAV1 facilitates lipid storage and mobilization in adipose tissues.

It was previously reported that CAV1 expression in human adipose tissue is up-regulated in obesity and obesity-associated type 2 diabetes [58]. However, this finding is converse to a report by Fernandez-Real et al. [10], who observed a major decrease in CAV1 expression in visceral adipose tissue of obese patients. In addition, as other studies have shown that insulin receptor expression and signaling is dependent on CAV1, increased expression of CAV1 in diabetic patients and mouse models may indicate an attempt to improve signaling under conditions of increased insulin resistance [58]–[60]. Meanwhile, CAV1 deficiency also leads to obesity accompanied by abnormalities in lipid metabolism, insulin resistance, hypertriglyceridemia, and dysregulated non-shivering thermogenesis [58].

Resistance to diet-induced obesity is one of the phenotypes of *Cav*1-null mice [17]. Obesity resistance in these mice can be attributed to the inability to convert TG in lipoprotein form to TG in lipid droplet form in WAT [17] in addition to the greater thermogenic capacity of BAT [61]. Any defect in the transport and/or storage of fatty acids or cholesterol in adipocytes of *Cav1*-null mice could lead to the alteration of lipid homeostasis, resulting in a lean body phenotype [61].

E2 has been proven to have stimulatory effects on HSL, resulting in increased basal lipolysis [62]. On the other hand, DHT has shown inhibitory effects on HSL in human subcutaneous WAT [63]. Similarly, these two sex hormones have opposite effects on UCP1 expression [64], [65]. To corroborate the previous results of other investigators, we examined sex-dependent HSL expression in response to treatment with sex hormones and HFD.

Importantly, expression levels of HSL and UCP1 were very similar with that of CAV1, especially in abdominal WAT and BAT, showing the same blunt regulation in HFD males (Figure 4). However, from the current data we are unable to conclude that E2 treatment in HFD-fed males has blunt effect on the expression levels of target proteins. But this similar expression patterns llowed us to hypothesize that CAV1 may have strong association with mitochondrial fatty acid oxidation and thermogenesis. To test this, we conducted Co-IP of CAV1-associated proteins and identified mitochondrial proteins in BAT, including ATP5B, ETF, ACAA2, and ACO2 (Figure 6). ATP5B and ETF are immensely important proteins in terms of energy expenditure, whereas ACAA2 and ACO2 are involved in fatty acid oxidation and the TCA cycle in mitochondria. Moreover, ACAA2 is a mitochondrial enzyme that catalyzes the last step of fatty acid oxidation, resulting in the release of acetyl CoA for the Kreb’s cycle as well as other oxidative enzymes such as CPT1 [66]. ACO2 plays a role in converting citrate to isocitrate as well as fatty acid oxidation products to substrate for the Kreb’s cycle, and it is reportedly down-regulated in HFD-induced obesity [67]. Furthermore, the immunoblot results for ATP5B and ETF demonstrated similar expression patterns as CAV1 mainly in abdominal WAT and BAT, suggesting concrete interactions among these proteins.

Recent reports demonstrated on the presence of soluble CAV1 in the cytoplasm and various other cellular compartments such as mitochondria [38], [68], attenuation of metabolic flexibility due to mitochondrial dysfunction in *Cav1* KO mice [7], and the positive role of E2 in mitochondrial biogenesis and signaling [69], supporting a novel connection between CAV1 and mitochondrial signaling. Here, our study showed that CAV1 interacts with major mitochondrial fatty acid oxidative and electron transport chain proteins. Additionally, similarity in the expression levels of CAV1, HSL, and UCP1 as well as novel mitochondrial partner proteins identified by Co-IP in E2-treated groups indicate a strong positive connection between CAV1 and the effects of E2 on mitochondrial function. These novel interactions between CAV1 and mitochondrial matrix proteins may function synergistically with inhibition of cholesterol accumulation at the mitochondrial membrane induced by CAV1 [70]. In this sense, the increased expression of both CAV1 and interacting mitochondrial proteins stimulated by E2 may cooperate with classical receptor-dependent stimulation of mitochondrial activity also induced by E2, resulting in sex hormone-dependent regulation of adipose tissue metabolism.

We further observed that BAT weight was reduced in DHT-treated groups, whereas it increased in all E2-treated groups except HFD-fed males. Interestingly, this regulatory pattern resembles the expression patterns of CAV1 and UCP1 in BAT. In addition, increased expression of UCP1 and CAV1 in BAT of E2-treated rats may reveal an essential connection between CAV1 and UCP1 for proper non-shivering thermogenesis *via* a modulatory effect on lipid mobilization [9], [71].

Although previous studies have indicated that *Cav1* KO mice are resistant to HFD-induced obesity [4], there is a controversy concerning the role of CAV1 in lipogenesis [10]. However, there are no reports on the effects of *Cav1* KD on the expression of major lipogenic genes or proteins in either 3T3-L1 or HIB1B adipocytes. In this regard, we report, for the first time, the up-regulation of major lipogenic and adipogenic genes as well as proteins in *Cav1* KD 3T3-L1 and HIB1B adipocytes (Figure 8).

It has been demonstrated that CAV1 directly interacts with both estrogen and androgen receptors [33], [72]. In this context, our *in vitro* study observed attenuated expression of ERα and AR in both adipocyte cell types, whereas ERβ expression was ameliorated only in 3T3-L1 adipocytes. These expression patterns suggest an intense connection with sex hormone receptors, especially ERα and AR (Figure 8). Moreover, CYP19 showed opposite expression patterns between the two adipocytes. An earlier report demonstrated that aromatase expression increases during differentiation of 3T3-L1 adipocytes [73]. In this context, the present study showed that *Cav1* KD cells have higher differentiation and adipogenic capacity, resulting in stimulation of CYP19 in 3T3-L1 adipocytes but not HIB1B cells (Figure 8). Under the present circumstances, we are not able to interpret the opposite effects of *Cav1* KD on aromatase expression in 3T3-L1 and HIB1B adipocytes. Further studies are necessary to directly address this result.

ERα is regarded as the one of the main targets responsible for the anti-obesity effects of E2 based on its expression levels in human subcutaneous WAT [74] as well as the results of a study using E2 inhibitor [75]. The present study supports these results, as exogenous E2 led to increased expression of ERα for the stimulation of estrogenic signaling in normal SD rats. Schlegel et al. (1999) identified CAV1 as a potential regulator of ERα signal transduction by observing ligand-independent translocation of ERα into the nucleus upon co-expression of CAV1 and ERα. Conversely, the role of ERβ in the development of obesity remains controversial [75], [76]. A recent report demonstrated that ERβ agonist treatment reduces adipogenic potential *via* negative crosstalk with PPARγ in female OVX Wistar rats [24]. In this study, abdominal WAT of E2-treated groups showed higher expression of ERβ, with females more prominently affected. However, in the case of BAT, stimulation of ERβ expression was observed only in females, which in turn may result in negative crosstalk of PPARγ-dependent lipogenic potential in these groups [21]. In case of BAT as well as gonadal and inguinal adipose tissues of male groups, ERβ failed to show any distinct pattern, suggesting a specific differential expression pattern for adipose tissues (Figure 5).

Androgen receptor (AR) is another important regulatory point of metabolism and body fat distribution. AR KO mice show late onset of obesity despite ameliorated lipolysis and UCP1 expression [77]. However, a recent report demonstrated that adipose tissue-specific AR KO mice undergo significant body weight gain when fed a HFD [78]. In this regard, higher expression of AR in adipose tissues of E2-treated rats indicates a positive role for AR in body weight reduction in SD rats. On the contrary, DHT treatment previously led to reduce AR and HSL expression in subcutaneous adipose tissue [63]. We also found either no significant effect in most groups or reduced expression of AR especially in HFD-fed female groups of WAT. Taken together, reduced expression of AR along with reduced expression HSL by DHT treatment may have contributed negatively in body weight.

Earlier reports have demonstrated that aromatase (CYP19) KO mice as well as humans with polymorphisms in the *Cyp19* gene exhibit endocrine imbalance and obesity [79], [80]. In addition, CYP19 in adipose tissue plays a pivotal role in circulating estrogen levels and fat distribution in males and postmenopausal females [76]. We propose that the opposite effects of E2 and DHT on CYP19 protein expression levels in WAT and BAT are strongly connected with the maintenance of a steady E2 concentration through regulation of estrogen synthesis. This differential regulation of CYP19 may regulate the availability of sex hormones during adipose tissue metabolism.

**Figure 9:**
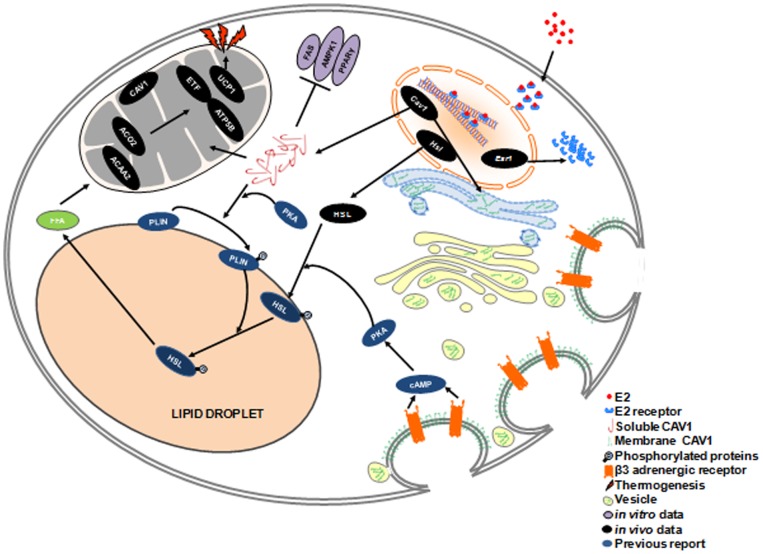
Possible roles of CAV1 in E2-dependent regulation of adipocyte metabolism.

Taken together, we describe the possible roles of CAV1 in E2-stimulated adipocytes conferring anti-obesity effects in Figure 9. The roles of DHT are not depicted as they were found to be the opposite in all metabolic events. E2 can effectively diffuse through the plasma membrane, subsequently binding with E2 receptors (ERs) in the cytosol. This results in transport of ERs into the nucleus, which increases the expression of *Cav1*, *Hsl*, and *Esr1*. Higher levels of ERα may be involved in the exponential stimulation E2-dependent signaling, whereas elevated HSL levels may indicate higher lipolytic capacity. Importantly, E2 highly stimulates two CAV1 pools using transcriptional and translational mechanisms. The first pool of CAV1 is transported to the plasma membrane through the endoplasmic reticulum and golgi bodies, whereas the second pool of CAV1 may directly transported to mitochondria from cytoplasm.

CAV1 in the plasma membrane is associated with β_3_-adrenergic receptor-dependent lipolysis. *Cav1*-ablated mice show attenuated β_3_-AR dependent lipolysis [71], and β_3_-AR is oppositely regulated by treatment with E2 versus DHT [20]. Therefore, higher CAV1 expression and E2 activity act synergistically to stimulate β_3_-AR signaling for the efficient phosphorylation of up-regulated HSL and peripilin (PLIN) by activated protein kinase A (PKA), thereby facilitating stimulated lipolysis [2]. CAV1 is known to be positively associated with phosphorylation of PLIN on lipid droplets [2]. In addition, Brasaemle et al. [81] reported the presence of CAV1 only in oil droplets of β_3_-AR stimulated cells by proteomic analysis. In this sense, we propose that higher levels of cytoplasmic CAV1 may stimulate phosphorylation of PLIN to amplify lipolysis in association with elevated β_3_-AR signaling induced by higher CAV1 levels in the plasma membrane.

Interestingly, our data suggest that CAV1 interacts with major mitochondrial resident proteins, which means a potent role for CAV1 in mitochondrial fatty acid oxidation and the electron transport chain resulting in elevated energy production. These elevated energy levels can be utilized by the higher amounts of UCP1 induced by E2, resulting in higher non-shivering thermogenenis or energy dissipation through heat (Figure 9).

In conclusion, CAV1 play important roles in adipose accumulation and is an important target protein for the hormonal regulation of adipose tissue metabolism by manipulating sex hormone receptors and mitochondrial oxidative pathways. Here, we elucidated, for the first time, the molecular mechanism underlying the effects of sex steroid hormones on sex-dimorphic regulation of CAV1. Based on current data, we postulate that *Cav1* deficiency not only results in leanness found in *Cav1*-deficient rodents but also followed by impaired sex hormone-dependent metabolic regulations in adipose tissue, thereby leading to abnormalities in lipid metabolism, insulin resistance, hypertriglyceridemia, and dysregulated non-shivering thermogenesis.

## Supporting Information

Figure S1 **Effects of sex hormone treatment on plasma levels of triglycerides (TG), free fatty acids (FFA), estradiol (E2), and dihydrotestosterone (DHT).**
(TIF)

Figure S2 **Corresponding control (β-actin) bands of each tissue that were used for normalization of western blot images.**
(TIF)

Table S1 **Statistical analysis of adipose tissue weight.** Adipose tissue weight between control and hormone treated groups were calculated by Student’s *t*-test, where, *p<0.05, **p<0.01. The significance of the effects of sex, diet and sex*diet were tested using multivariate ANOVA (M-ANOVA), where NS represents a *p*>0.05.
(DOCX)

Table S2 **Statistical analysis of adipocyte area (abdominal WAT) or lipid area (BAT) in** **Figure 2** **of main manuscript.** Adipocyte area (abdominal WAT) or lipid area (BAT) between control and hormone treated groups were calculated by Student’s *t-*test, where, **p<0.01. The significance of the effects of sex, diet and sex*diet were tested using multivariate ANOVA (M-ANOVA), where NS represents a *p*>0.05.
(DOCX)
