# Supplementary figures and images for: Sex-Dependent Expression of Caveolin 1 in Response to Sex Steroid Hormones Is Closely Associated with Development of Obesity in Rats
Source: PLoS One. 2014 Mar 7;9(3):e90918. doi: 10.1371/journal.pone.0090918 (PMC3948350; doi:10.1371/journal.pone.0090918)

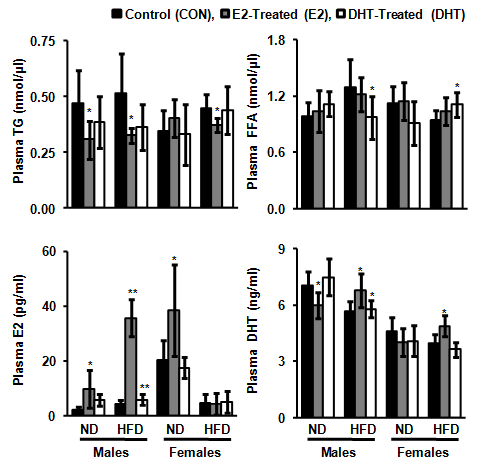

Supplement: Figure S1 — Effects of sex hormone treatment on plasma levels of triglycerides (TG), free fatty acids (FFA), estradiol (E2), and dihydrotestosterone (DHT). (TIF) [file pone.0090918.s001.tif]

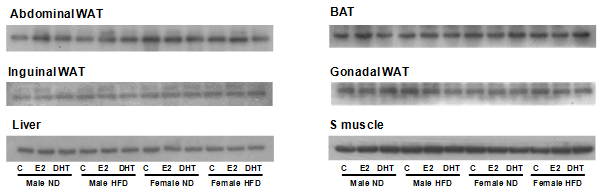

Supplement: Figure S2 — Corresponding control (β-actin) bands of each tissue that were used for normalization of western blot images. (TIF) [file pone.0090918.s002.tif]
