# Supplementary material for: Sex-Dependent Expression of Caveolin 1 in Response to Sex Steroid Hormones Is Closely Associated with Development of Obesity in Rats
Source: PLoS One. 2014 Mar 7;9(3):e90918. doi: 10.1371/journal.pone.0090918 (PMC3948350; doi:10.1371/journal.pone.0090918)
Supplement: Table S1 — Statistical analysis of adipose tissue weight. Adipose tissue weight between control and hormone treated groups were calculated by Student’s t-test, where, *p<0.05, **p<0.01. The significance of the effects of sex, diet and sex*diet were tested using multivariate ANOVA (M-ANOVA), where NS represents a p>0.05. (DOCX) [file pone.0090918.s003.docx]

|  |  | **Male** | | **Female** | | ***p* values** | | |
| --- | --- | --- | --- | --- | --- | --- | --- | --- |
| **Tissue** | **Group** | **ND** | **HFD** | **ND** | **HFD** | **Sex** | **Diet** | **Sex*Diet** |
| Abdominal WAT Weight(g) | Con | 2.38± 0.39 | 5.41±1.17 | 1.53±0.57 | 1.78±0.62 | <0.01 | <0.01 | <0.01 |
|  | E2 | 0.87±0.031** | 1.24±0.43** | 0.61±0.25* | 0.59±0.24* | <0.01 | NS | NS |
|  | DHT | 2.65 ±0.57 | 5.94±0.63 | 1.13±1.75 | 1.75±0.34 | <0.01 | <0.01 | <0.01 |
| Gonadal WAT Weight(g) | Con | 3.43± 0.48 | 5.74±0.96 | 1.62±0.65 | 4.75±0.91 | <0.01 | <0.01 | NS |
|  | E2 | 1.82±0.35** | 2.12±0.36** | 1.33±0.73 | 1.75±0.55** | NS | NS | NS |
|  | DHT | 3.52 ±0.42 | 6.81±0.93 | 2.62±0.35* | 4.77±0.64 | <0.01 | <0.01 | NS |
| Inguinal WAT Weight(g) | Con | 0.76±0.12 | 1.29±0.36 | 0.62±0.15 | 0.62±0.1 | <0.01 | <0.05 | <0.05 |
|  | E2 | 0.42±0.042** | 0.39±0.09* | 0.29±0.107* | 0.31±0.12 | NS | NS | NS |
|  | DHT | 0.76±0.18 | 0.5±0.26 | 0.37±0.08* | 0.83±0.21 | NS | NS | <0.05 |
| BAT Weight | Con | 0.45±0.07 | 0.65±0.1 | 0.29±0.03 | 0.34±0.024 | <0.01 | <0.01 | <0.05 |
|  | E2 | 0.48±0.028 | 0.44±0.06* | 0.34±0.029* | 0.37±0.088 | <0.01 | NS | NS |
|  | DHT | 0.32±0.07* | 0.42±0.09* | 0.27±0.02 | 0.27±0.039* | <0.01 | NS | NS |
