# Supplementary material for: Sex-Dependent Expression of Caveolin 1 in Response to Sex Steroid Hormones Is Closely Associated with Development of Obesity in Rats
Source: PLoS One. 2014 Mar 7;9(3):e90918. doi: 10.1371/journal.pone.0090918 (PMC3948350; doi:10.1371/journal.pone.0090918)
Supplement: Table S2 — Statistical analysis of adipocyte area (abdominal WAT) or lipid area (BAT) in Figure 2 of main manuscript. Adipocyte area (abdominal WAT) or lipid area (BAT) between control and hormone treated groups were calculated by Student’s t-test, where, **p<0.01. The significance of the effects of sex, diet and sex*diet were tested using multivariate ANOVA (M-ANOVA), where NS represents a p>0.05. (DOCX) [file pone.0090918.s004.docx]

|  |  | **Male** | | **Female** | | ***p* values** | | |
| --- | --- | --- | --- | --- | --- | --- | --- | --- |
| **Area** | **Group** | **ND** | **HFD** | **ND** | **HFD** | **Sex** | **Diet** | **Sex*Diet** |
| Adipocyte area Abdominal WAT(mm^2^) | Con | 0.081±0.014 | 0.186±0.049 | 0.059±0.016 | 0.076±0.007 | <0.01 | <0.01 | <0.01 |
|  | E2 | 0.033±0.008** | 0.052±0.011** | 0.017±0.005** | 0.038±0.009** | <0.01 | <0.01 | NS |
|  | DHT | 0.094 ±0.016 | 0.15±0.028 | 0.067±0.014 | 0.084±0.02 | <0.01 | <0.01 | NS |
| Lipid area BAT(mm^2^) | Con | 0.0017±0.0003 | 0.010±0.0026 | 0.0062±0.0019 | 0.013±0.0061 | NS | <0.01 | NS |
|  | E2 | 0.001±0.0001** | 0.002±0.0005** | 0.0015±0.0002** | 0.003±0.001** | <0.01 | <0.01 | NS |
|  | DHT | 0.0021±0.0008 | 0.008±0.0012 | 0.0042±0.0013 | 0.012±0.0066 | NS | <0.01 | NS |
